# Ultrastructure of dorsal root ganglia

**DOI:** 10.1007/s00441-023-03770-w

**Published:** 2023-04-20

**Authors:** Rainer Viktor Haberberger, Jaliya Kuramatilake, Christine M Barry, Dusan Matusica

**Affiliations:** 1 Division of Anatomy and Pathology, School of Biomedicine, The University of Adelaide, Adelaide, Australia; 2 Anatomy, Histology & Pathology, College of Medicine and Public Health, Flinders University, Adelaide, Australia

**Keywords:** Spinal ganglia, Neuron subtypes, Organelles, TEM, SEM

## Abstract

Dorsal root ganglia (DRG) contains thousands of sensory neurons that transmit information about our external and internal environment to the central nervous system. This includes signals related to proprioception, temperature, and nociception. Our understanding of DRG has increased tremendously over the last 50 years and has established the DRG as an active participant in peripheral processes. This includes interactions between neurons and non-neuronal cells such as satellite glia cells and macrophages that contribute to an increasingly complex cellular environment that modulates neuronal function. Early ultrastructural investigations of the DRG have described subtypes of sensory neurons based on differences in the arrangement of organelles such as the Golgi apparatus and the endoplasmic reticulum. The neuron-satellite cell complex and the composition of the axon hillock in DRG have also been investigated, but, apart from basic descriptions of Schwann cells, ultrastructural investigations of other cell types in DRG are limited. Furthermore, detailed descriptions of key components of DRG, such as blood vessels and the capsule that sits at the intersection of the meninges and the connective tissue covering the peripheral nervous system, are lacking to date. With rising interest in DRG as potential therapeutic targets for aberrant signalling associated with chronic pain conditions, gaining further insights into DRG ultrastructure will be fundamental to understanding cell–cell interactions that modulate DRG function. In this review, we aim to provide a synopsis of the current state of knowledge on the ultrastructure of the DRG and its components, as well as to identify areas of interest for future studies.

## Introduction

The structure and ultrastructure of the dorsal root ganglia (DRG) have been investigated and described from the advent of histological and electron microscopical studies. The father of modern neuroscience, Ramon y Cajal, using two of the most powerful microscopes available at the time, prepared breakthrough drawings of DRG and defined the morphological characteristics of individual DRG neurons from his observations at the end of the nineteenth century (Garcia-Poblete et al. 2003).

Histology and the concomitant developments in microscopy with the use of precision engineering techniques and mass manufacture led to increasingly precise descriptions of tissues and cells. Ernst Ruska at the University of Berlin along with Max Knoll built the first transmission electron microscope (TEM) in 1931 that surpassed the optical limitations of light microscopy earning Ruska the Nobel Prize for Physics in 1986. With this technology, the inner structure of cell organelles, including the cytoskeleton and membranes, became accessible for detailed morphological investigations. Consequently, the ultrastructure of DRG was studied using electron microscopy soon after methods for TEM investigations were fully established and accessible to researchers (Beams et al. 1952; Hess 1955).

The fine structure of DRG neurons was first published in the early 1950s, but unfortunately, the images lacked the required resolution for researchers to draw clear and comprehensive conclusions about the structure of organelles present in neurons (Beams et al. 1952; Hossack 1954). A few years later, after the technical challenges of electron microscopy were solved, Palay and Palade (1955) and Cervos-Navarro (1959) demonstrated the lipid bilayer in the nuclear membrane, mitochondria, smooth and rough endoplasmic reticulum, ribosomes, and neurofilaments in rat DRG neurons. These features have been described as characteristic of sensory neurons. Those early findings and descriptions were the foundation for a broader understanding of the functions of organelles present in neurons. This was the time that the structure of DNA had just recently been proposed, and the Nissl substance was observed in neurons. The latter has been identified as rough endoplasmic reticulum and cytoplasmic polyribosomes but was then thought to be an artefact of fixation. However, Andres (1961a) reviewed the ultrastructure of DRGs and associated neurons in 1961 and expanded the existing knowledge with new data that described the capsule, endoneurium, and satellite cells and provided the first description of different types of DRG neurons at the ultrastructural level. Subsequently, generations of researchers have investigated the ultrastructure of animal and human DRGs and their resident cells using techniques ranging from conventional TEM and scanning electron microscopy (SEM) to newer methods, such as cryo-electron tomography. They provided three-dimensional visualisation of DRG components at nanometre resolution with minimal processing-associated distortion (Shahmoradian et al. 2014). Scanning EM investigations have shown the developmental transformation of DRG neurons from bipolar into pseudo-unipolar cells, their use of phagocytosis to ingest and eliminate particles, the morphology of neuronal processes, axonal glomeruli, and neuron-satellite cell clusters within DRG (Bowen et al. 2007; Connolly et al. 1985; Haider et al. 1983; Matsuda et al. 1996, 2005; Matsuda and Uehara 1981).

The focus of this review is on the ultrastructure of the cellular components of DRGs, starting with sensory neurons and their organelles and expanding to other cell types, followed by a description of the connective tissues of the capsule and blood vessels.

## Structure of DRG neurons

A DRG contains an accumulation of cell bodies of thousands of sensory neurons, which form a swelling in the dorsal root of a spinal nerve. The number of neurons per DRG has been shown to vary across spinal segments, correlating with DRG size, and vary across species, and has been shown to range from 70,000 neurons in young human to 15,000 in rats (Nagashima and Oota 1974; Tandrup 2004; West et al. 2012). The cell bodies of DRG neurons, at least in humans, rats, mice, and frogs, are predominantly located in the outer marginal area of the ganglion, near the capsule, while the centre is occupied by bundles of nerve fibres and scattered individual neurons (Andres 1961a; Haberberger et al. 2019; Matsumoto and Rosenbluth 1985). The neuronal cell bodies represent pseudo-unipolar primary afferent neurons, containing a stem axon that divides into two branches. One branch innervates peripheral targets, and the other terminates in the dorsal horn of the spinal cord. Human adult DRG neuron somata are large, ranging between 20 and 120 µm in diameter, with a smaller average size in infants (Nagashima and Oota 1974).

The easy accessibility via dissection and the relatively large size of the cells made DRG interesting for early microscopical investigations, and by 1955, the ultrastructure of DRG neurons had been investigated and reviewed (Dawson et al. 1955; Hess 1955; Palay and Palade 1955). Andres (1961a, b) further expanded the ultrastructural investigations from sensory neurons to many key cells and components of rat DRG (Andres 1961a). Scanning electron microscopy investigations of human DRG yielded high-resolution three-dimensional information (Matsuda and Uehara 1981; Siew 1979), and they confirmed the presence of neuronal somata with unipolar processes at the margins of the ganglion, while nerve fibre bundles were primarily located in the centre (Siew 1979). The spatial location of the somata and the general architecture of DRG appear to be the same at different vertebral levels. Where ultrastructural differences do exist within individual ganglia, they are primarily observed in soma size and the organisation of subcellular structures, which manifest the differences in organelle density, and render the appearance of the structures as light or dark.

## Organelles in DRG neurons

Sensory DRG neurons possess large cell bodies that contain the key organelles such as the nucleus and Golgi apparatus embedded in the cytoplasm and surrounded by a plasma membrane with a thickness of about 10–13 nm (Andres 1961a). Interestingly, even though the cell bodies of DRG neurons are large, the majority of the cytoplasm together with mitochondria and endoplasmic reticulum are present in the long axonal processes of these neurons (Devor 1999).

### Nucleus/nucleolus

The cell nucleus contains the DNA, multiple forms of RNA, and a plethora of proteins that are involved in transcription, forming of the chromatin structure, or the transport of proteins and RNAs in and out of the organelle (Figs. 2 and 3). In DRG neurons, nuclei are situated paracentral but at least 5–7 μm away from the neuronal process or plasma membrane (Andres 1961a; Bunge et al. 1967). The size of the nucleus usually reflects the size of a cell. The nuclei of DRG neurons in rats are usually between 12 and 22 μm in diameter with an electron-lucent nucleoplasm. Rat, bovine, and dog nuclei are similar in size (Andres 1961a; Fadda et al. 2016; McCracken and Dow 1973; Mitro et al. 1983; Tongtako et al. 2017). Large and small neurons have nuclei that are often elliptical and round in shape, respectively (Andres 1961a). The nuclei are enclosed by a nuclear membrane that contains nuclear pores to enable transport into and out of the nucleus. The diameter of the pores ranges from 30 to 100 nm, with nuclear pores in the outer membrane wider (70–100 nm) than those in the inner membrane (30–50 nm) (Andres 1961a; Bunge et al. 1967; McCracken and Dow 1973) (Table 2). A space of 10–25 nm (in dogs 17–45 nm) in width separates the inner and outer nuclear membrane and forms the perinuclear space (Mitro et al. 1983) which is also referred to as “the circumnuclear cistern of Watson” (Andres 1961a; Cervos-Navarro 1959; Palay and Palade 1955). The nuclei of all investigated species including humans (Berciano et al. 2007) usually possess a single eccentrically located nucleolus. In dogs, sometimes two nucleoli have been observed in small-sized sensory neurons (Andres 1961a; Mitro et al. 1983). The size of nucleoli varies, and their matrix contains plates of densely packed chromatin and fibrillary and chromatin-associated structures in the light areas (karyosomes) (Fig. 3). A distinct component present in nuclei was first described by Cajal in 1903 and named Cajal bodies. These structures are usually 0.5–2.4 μm in size and involved in the production of small nuclear and nucleolar RNAs (snRNAs, snoRNAs) (Berciano et al. 2007; Neugebauer 2017). The number of nuclear Cajal bodies in most non-neuronal cell types has been described as 1–4 per cell, while neurons of the human DRG were shown to contain up to 20 per cell (Table 2) (Berciano et al. 2007). Ultrastructure investigations of the Cajal bodies have demonstrated a granular appearance, and immunogold-electronmicroscopy has indicated the presence of the proteins coilin, fibrillarin, and SMN protein (Berciano et al. 2007).

### Endoplasmic reticulum (ER)

The cytoplasm of DRG neurons is filled with reticular arrangements of smooth tubules and cisterns, the smooth endoplasmic reticulum (ER) (Palay and Palade 1955), ribosome-rich rough ER, and free ribosomes (Nissl substance, tigroid) (Andres 1961a; Dawson et al. 1955; Johnson and Sears 2013; McCracken and Dow 1973; Palay and Palade 1955; Philippe and Droz 1988). The ER is the largest organelle and is found to be a prominent structure throughout the cell body. It consists of tubular membranous structures with a membrane thickness of 6–7 nm and a lumen of 30–250 nm in diameter (Table 2). They are often found to be oriented parallel and separated at intervals of 80 and 200 nm (Anderson and Van Breemen 1958; Cervos-Navarro 1959; Palay and Palade 1955). The ER is continuous with the nuclear membrane and in larger sensory neurons, almost spans the area between nucleus and plasma membrane (Cervos-Navarro 1959). Sensory DRG neurons have a high protein turnover due to the length of their process and related homeostatic processes, such as maintenance of cell membrane structure and the regulation of membranous receptor proteins. Thus, the cytoplasm of DRG neurons is rich in ER networks. Different DRG neuron subtypes show variations in the abundance and the arrangement of the ER in the cytoplasm. In larger DRG neurons the ER occasionally forms a ring-like structure surrounding the nucleus (Bunge et al. 1967). Furthermore, spaces between accumulations of rough ER that contain collections of mitochondria are referred to as “cytoplasm streets” (Table 1). Ribosomes (earlier described as Palade granules) have been described as rod-like particles, 25–30 nm in diameter (Palay and Palade 1955), located either on the cytoplasmic side of the ER or in the cytoplasm as polyribosome rosettes (Table 2) (Andres 1961a; Bunge et al. 1967; Cervos-Navarro 1959; McCracken and Dow 1973; Palay and Palade 1955). Smooth ER has been identified in studies of rat and bullfrog DRG and was described as having a similar appearance to the rough ER, but tighter packing, narrow cisterns, and absence of ribosomes (Lindsey et al. 1981; Palay and Palade 1955; Palay and Wissig 1953; Rambourg et al. 1983).

**Table 1:** Classification of DRG neurons based on the ultrastructure

	Andres (1961a)	Duce and Keen (1977)	Rambourg et al. (1983)	Sommer et al. (1985)	Phillipe and Droz (1988)
	Rat	Rat	Rat	Mouse	Chick
A	A_1_ • Large cells (40–75 μm) • Usually, one nucleolus • Even distribution of Nissl bodies (rER) in the cytoplasm • Golgi apparatus extends from nucleus to cell membrane • Initial segment glomerulus-like structure • Myelinated process originates	A_1_ • High proportion of neurofilaments • Golgi bodies scattered throughout the cytoplasm • Golgi apparatus has 3–4 lamellae • Two types of mitochondria • Type 1 conventional • Type 2 smaller with different arrangement of cristae	A_1_ • Large cells (40–75 μm) • Even distribution of Nissl bodies (rER) in the cytoplasm • Short stacks of Golgi saccules • Rod-like mitochondria	Aα • Large cells (30–50 μm) • rER present throughout the soma but predominantly in outer part of soma • No carbonic anhydrase activity in soma, high activity in satellite cell • Moderate uptake of [3H]L-glutamine • No clear “zonation” of organelles	A1 • Large cells • Abundance of rER present throughout the soma • Tiny bundles of neurofilaments • Small groups of Golgi • Calbindin immunoreactive
	A_2_ • Large cells (about 45 μm) • Usually, one nucleolus • Large light cytoplasm channels between accumulations of Nissl substance (rER) in the cytoplasm • Initial segment glomerulus-like structure • Thick, myelinated process originates	A_2_ • Lower proportion of neurofilaments running in strands • “Islands” of ribosomes • Compared to A1 larger, more complicated Golgi complexes • Golgi apparatus has 6–8 lamellae • More elongated mitochondria	A_2_ • Nissl bodies (rER) separated by cytoplasm streets • Neurofilaments running in strands in cytoplasm streets • Golgi bodies smaller compared to A_1_	Aβ • Large cells (30–50 μm) • rER present throughout the soma. Short rER cisternae • Wide channels of neuroplasma, enriched in neurofilaments • Moderate carbonic anhydrase activity in soma, high activity in satellite cell • High uptake of [3H]L-glutamine • No clear “zonation” of organelles	A2 • Large cells • sER present next to the membrane • Wide channels of neuroplasma, enriched in neurofilaments • Mitochondria close to rER • No clear “zonation” of organelles • No calbindin immunoreactivity
	A_3_ • Small cell size similar to B group • Usually, one nucleolus • Light cytoplasm • Condensed zones of rER • No prominent glomerulus-like structure initial segment • Axons are 2–3-μm thick • Myelinated process	A_3_ • Lower proportion of neurofilaments • Abundant rER • Elongated Golgi stacks • Numerous round or ovoid mitochondria	A_3_ • Size closer to B type cells but no zonation of organelles • Nissl bodies (rER) scattered throughout the perikaryon separated by cytoplasm • Golgi complexes are predominantly present perinuclear	Aγ • Large cells (30–50 μm) • rER present throughout the soma. Elongated rER cisternae • Strong carbonic anhydrase activity in soma, high activity in satellite cell • No uptake of [3H]L-glutamine • No clear “zonation” of organelles	
	B_1_ • Small cell size (25–40 μm) • Often 2 nucleoli • Dark cytoplasm • Condensed zones of rER • rER accumulation smaller compared to A group • No clear evidence of Initial segment glomerulus-like structure • Golgi apparatus perinuclear • Unmyelinated or thinly myelinated process • Adjacent satellite cells have contact with processes of other cells	B_1_ • rER present in the periphery of the cell parallel to cell membrane • Perinuclear Golgi apparatus • Large and numerous Golgi stacks • Numerous mitochondria • Numerous dense bodies B_1▢_ • Abundant rER • Golgi complexes large and scattered throughout the cytoplasm B_1b_ • Less rER compared to B_1_ • Golgi complexes perinuclear and more strongly curved • Outer laminae of Golgi complexes dilated • Mitochondria shorter compared to B2	B_1_ • Larger than B2 cells • Cortical arrangement of rER • Perinuclear Golgi complexes • Extensive network of perinuclear sER? (tubular ER cisternae)	Bα • Small cells (20–35 μm) • rER predominant in the outer part of the soma • Golgi apparatus in the mid zone of the soma and acid phosphatase positive • Mitochondria predominantly close to the nucleus • No carbonic anhydrase activity in soma *• NO carbonic anhydrase activity in satellite cells* • High uptake of [3H]L-glutamine • Clear “zonation” of organelles	B1 • Smaller in size compared to A neurons • rER predominant in the outer part of the soma • This zone was followed by a clear Golgi-zone and • An innermost perinuclear zone occupied by mitochondria and neurofilaments • Calbindin-immunoreactive
	B_2_ • Small cell size (18–30 μm) • Often 2 nucleoli • Dark cytoplasm in the centre of the cell • Condensed zones of rER in the periphery of the perikaryon	B_2_ • Organelles distributed evenly • Golgi bodies small with low numbers • Mitochondria ovoid with transverse cristae	B_2_ • Smallest B type cell • Clear zonation • Cortical zone rER • Perinuclear (juxtanuclear) zone Golgi complexes and abundant mitochondria and sER	Bβ • Small cells (20–35 μm) • rER predominant in the outer part of the soma • Golgi apparatus in the *perinuclear zone* of the soma and acid phosphatase positive • Mitochondria predominantly close to the nucleus • No carbonic anhydrase activity in soma *• Strong carbonic anhydrase activity in satellite cells* • High uptake of [3H]L-glutamine • Clear “zonation” of organelles	B2 • rER predominant in the outer part of the soma • A belt of in the mid- and inner area of the soma • Not as prominent “zonation” compared to B1 • No calbindin immunoreactivity
	B_3_ • Small cell size (< 18 μm) • Often 2 nucleoli • Dark cytoplasm • Condensed zones of rER • rER accumulation smaller compared to A group • Adjacent satellite cells have contact with processes of other cells	B_3_ • Small cell size • Abundant rER • Large nucleus • Large Golgi bodies		Bγ • Small cells (20–35 μm) • rER predominant in the outer part of the soma • Golgi apparatus in the mid zone of the soma and acid phosphatase positive • Mitochondria *and large dense core vesicles* predominantly close to the nucleus • No carbonic anhydrase activity *and no acid phosphatase activity* in soma *• Substance P present* • High uptake of [3H]L-glutamine • Clear “zonation” of organelles	
			C • Smallest ganglion cell type • Small Nissl bodies in the middle zone with mitochondria and lysosomes • Perinuclear Golgi complexes	C • Very small cells (less 20 μm) • rER throughout the soma • Only few Golgi apparatus profiles • Mitochondria *were elongated* • No carbonic anhydrase activity *and no acid phosphatase activity* in soma • Strong carbonic anhydrase in satellite cells *• Substance P present* • High uptake of [3H]L-glutamine • Clear “zonation” of organelles	

Golgi apparatus = entire Golgi system of a cells

Golgi stack = individual stack of cisternae

**Table 2:** Presence and size of organelles in DRG neurons and satellite cells

**Organelle**	**DRG neuron**	**Satellite cell**
Cell membrane	10–15 nm	7–12 nm
Nuclei	Ovoid 12–22 μm	Round-oval 4–8 μm
Nucleolus	Usually 1, size varies	Present
Nuclear membrane	Inner membrane thicker compared to outer membrane, space in between 15 and 30 nm	Present
Nuclear pores	30–105 nm but different in size between inner and outer membrane	Present
Vesicles	25–60 nm	35–70 nm
Mitochondria	Round 0.5 × 2 μm or thick threads 4–5-μm long and 0.15–0.25-μm thick	Small, compact
Ribosomes	25–30 nm	Present
Neurofibrils/neurofibrillary tangles	6–10 nm/25 nm	Infrequent present
Golgi apparatus	Complexes 0.5–1.5 μm with 30–100 nm vesicles	Present
Endoplasmic reticulum	Membrane thickness 6–7 nm Lumen diameter 30–250 nm	Present
Pigment granules (lipofuscin)	Present, 0.2–1.5 μm, Membrane 10 nm	Present
Cajal bodies	Number can vary, 0.5–2.4 nm	

### Golgi-apparatus, multivesicular bodies and lysosomes

The Golgi-apparatus, first described by Camillo Golgi in 1898, is an organelle involved in protein modification, endocytosis, and targeted delivery. It was first named *apparato reticolare interno* and in ultrastructural investigations of DRG neurons appeared as large numbers of separated areas containing stacks of Golgi saccules (Cervos-Navarro 1959; McCracken and Dow 1973; Palay and Palade 1955). Saccules, tubules, and vesicles have sizes between 30 and 100 nm (Table 2). In rat DRG neurons, individual Golgi complexes reached a size of up to 2 μm (Figs. 2 and 3) (Koga and Ushiki 2006). The polarity of the Golgi apparatus is demonstrated by the cis, middle, and trans-sides. Scanning electron microscopy (EM) of rat DRG neurons showed 30-nm-wide fenestrations on the cis side, while the trans side demonstrated 100 nm fenestrations, tubular structures, and the close proximity to the rough ER (Koga ang Ushiki 2006). The Golgi apparatus in large light neurons spans from the nucleus to the plasma membrane whereas in small, dark cells is located near the nucleus (Andres 1961a). Smaller dark neurons can be identified in rodents as nociceptive neurons by labelling with isolectin B4. The enzymes galactosyltransferase (1,3GT) and isoglobotriaosylceramide synthase (iGb3S) are associated with the Golgi apparatus and form the binding partner for isolectin B4 (Fullmer et al. 2007).

Multivesicular bodies belong to the endosomal system and are involved in a variety of processes, such as internalisation, recycling, and degradation of membrane receptors and generation and release of extracellular vesicles (exosomes) (Piper and Katzmann 2007). Irregular-shaped multivesicular bodies are found in DRG neurons near Golgi stacks (Bunge et al. 1967; Palay and Palade 1955). The multivesicular bodies can contain different amounts of vesicles, patches of dense structures, and discs. Lysosomes, in early studies, were described as dense bodies and are predominantly found in the perinuclear cytoplasm (Bunge et al. 1967) (Figs. 2 and 3). They contain electron-dense molecules often in association with lipofuscin (Meier et al. 2020).

### Mitochondria

Dorsal root ganglion neurons have long processes and therefore a high protein turnover which is required for molecules to be produced and transported in sufficient quantities over long distances. Consequently, the energy consumption in DRG neurons is high, and they are dependent on mitochondrial glucose metabolism and fatty acids for ATP synthesis (Russell et al. 2002). Mitochondria are distinctive organelles that possess a double membrane and functionally specialized compartments. Studies on rabbit DRG neurons found mitochondria throughout the cell body and processes (Martinelli et al. 2006) (Figs. 2 and 3). The proportion of mitochondria increased with cell size during development but subsequently decreased with age (Ledda et al. 2001; Martinelli et al. 2006). Mitochondria in rat DRG neurons seemed to be present in two types: The “filament” type had the usual elongated shape, cristae mitochondriales and a matrix that is encapsulated by a smooth membrane. The other type was either rounded or irregularly shaped and had numerous invaginations and a low number of cristae (Andres 1961a; Cervos-Navarro 1959). In the slow loris, two forms of mitochondria, vacuolated and filamentous, were present in DRG neurons, with the vacuolated mitochondria predominantly present in small dark cells (Ahmed and Kanagasuntheram 1976). Mitochondria can change size and morphology according to the energy demand; thus, whether the above-described two forms represent two functional states of one type is not certain. The location of mitochondria is different in different types of neurons. They are evenly distributed in large light DRG neurons while located predominantly close to the nucleus of small dark neurons (Table 1) (Andres 1961a; Berthold 1966; Ledda et al. 2001).

### Microtubules and neurofilaments

Cell morphology and shape are modulated and maintained through the cytoskeleton. Well-defined cytoskeletal elements, such as microtubules and neurofilaments, are present in DRG neurons (van den Bosch de Aguilar and Goemaere-Vanneste 1984). Lighter areas of the cytoplasm contain parallel or irregularly arranged bundles of neurofibrils consisting of 6- to 10-nm-diameter neurofilaments. The bundles are usually dispersed between the Golgi saccules and the ER without a specific orientation (Andres 1961a; Cervos-Navarro 1959; Palay and Palade 1955). Neurofibrillary tangles in somata of rat sensory neurons have been described as paired helical filaments, and they change with increasing age (van den Bosch de Aguilar and Goemaere-Vanneste 1984). These helical structures can be up to 25 nm in diameter, with a single filament about 9 nm in diameter (van den Bosch de Aguilar and Goemaere-Vanneste 1984).

### Glycogen, pigments, and lipofuscin

Glycogen, an easily accessible source of energy, has been observed as aggregates of 19–35 nm in diameter in the cytoplasm of cultured embryonic DRG neurons near the plasma membrane (Bunge et al. 1967). Similar aggregates, 20–40 nm in diameter and occasionally surrounded by membranes, have been identified in the marginal areas and axon hillock of small sensory neurons of frogs (Berthold 1966). Studies in the 1950s identified membrane bound small vesicles of 20–50 nm in size. They were similar to vesicles found in synaptic endings (Cervos-Navarro 1959; Palay and Palade 1955), as well as pigment granules of 200–1500 nm in size, which were irregularly shaped with lighter areas or pigment granules that were round to elliptic in shape (Cervos-Navarro 1959). Lipofuscin is a component observed in the cell bodies of DRG neurons of all investigated species, including humans (Andres 1961b; Cervos-Navarro 1959; McCracken and Dow 1973; Schmidt et al. 1997). It is a pigment that contains cross-linked lipids, sugars, and proteins and has been described as osmiophilic lipofuscin granules (Andres 1961a). Cells cannot degrade lipofuscin; thus, it accumulates in neurons, including DRG neurons, over time (Moreno-Garcia et al. 2018; Schmidt et al. 1997). Lipofuscin-like granules are present in DRG neurons of all species investigated. Individual granules are surrounded by a membrane with a thickness of 10 nm and often contain dense material and a vacuole. Lamellar lipofuscin-like granules have been found in granules in small dark neurons in rats (Andres 1961a).

## Ultrastructure of axonal processes of DRG neurons

Sensory DRG neurons possess a pseudo-unipolar process, an axon that branches to form of a T-junction with a central process synapsing in the spinal cord dorsal horn and a peripheral process that ends in the target tissue (Nascimento et al. 2018). The axon begins with a specialized structure, the axon hillock, which is followed by the initial segment of the axon (Leterrier 2018). In most neurons, the axon hillock is key not only to the summation of synaptic inputs and the generation of action potentials, but also to the control of protein trafficking between the cell body and the axon (Leterrier 2018). The pseudo-unipolar neurons do not possess synapses in DRG, and hence action potentials are not generated. This represents a unique feature whose functions are not yet clearly understood. Recent studies on DRG of zebra fish showed that the arrangement of microtubules in both branches of the T-junction is arranged in a way that is characteristic for axons but not dendrites (Shorey et al. 2021). Axon hillock and T-junction of DRG neurons determine the passage of action potentials to the central terminals of sensory neurons (Sundt et al. 2015). The axon hillock contains neurofilaments, a large number of mitochondria, and occasionally vesicles with a diameter of 25–60 nm (Andres 1961a). Larger neurons appear to possess larger mitochondria in the axon hillock. Investigations using freeze-fracture and scanning electron microscopy of DRG neurons from frogs have shown that the axon hillock contains numerous mitochondria, microtubules, and neurofilaments, a high number of particles in the plasma membrane, but almost no ER (Matsumoto and Rosenbluth 1985).

The axon hillock together with initial glomerulum, the initial complex, and the first part of the axon have been described as initial complex (Murayama et al. 1991). The initial complex of the human DRG, here in particular of larger neurons, is occasionally surrounded by onion-like arrangement of layers of satellite cells (Murayama et al. 1991).

The glomerulum, axons of sensory neurons that coil as they emerge from the axon hillock, the initial glomerulum, was first described from Cajal in 1907. Comparative studies have shown that axonal glomeruli are present in rabbits and rats but no in frogs or chicks (Matsuda et al. 2005). In humans, the glomerulum has been identified as part of the initial complex (Murayama et al. 1991).

The axon initial segment is in most neurons the site of action potential generation. Sensory DRG neurons however generate, like many sensory neurons, the action potential at the peripheral ending. Nevertheless, spontaneous generation of action potentials at the initial segment of sensory neurons has been shown to be the underlying mechanism of spontaneous pain, a hallmark of neuropathic pain in mice (Nascimento et al. 2022, 2018). It has been described in mouse, rat, and human DRG neurons (Hedstrom et al. 2007; Murayama et al. 1991; Nascimento et al. 2022, 2018; Schmidt et al. 1997).

In rats, the axon has similar content to the axon hillock and at its myelin-free origin has a thickness of 2–10 μm (Andres 1961a). Ultrastructural analysis of neurites extending from DRG explants in response to exposure to notochordal and chondrocyte-like cells showed the presence of neurofilaments, microtubules, and vesicles in the cytosol. Neurites were in close contact with Schwann cells and had a diameter of approximately 500 nm (Larsson et al. 2012).

## Extrinsic neuronal processes in DRG

Sensory neurons in DRG do not possess dendrites and are believed to have no synaptic contacts with other neurons. However, the DRG can contain endings from neurons with origin outside the DRG. Scanning electron microscopy has shown the three-dimensional arrangement of extrinsic neurites and demonstrated the presence of pericellular nerve fibre baskets that had been first described by Dogiel and Ramon y Cajal in 1896 and 1907. These structures were named *pericellular* baskets (Matsuda et al. 2005; Matsuda and Uehara 1981). The pericellular baskets are present in rat and rabbit DRG but could not be found in bullfrog or chick DRG. These baskets have been associated with pathological states including models of nerve injury but are also found in DRG of healthy animals, though in very low numbers (Matsuda et al. 2005; Matsuda and Uehara 1981; Michael et al. 1999). Numbers of baskets increase in response to nerve damage. Pericellular baskets have been shown to consist of sympathetic and sensory fibres that sprout in response to nerve damage (Chung et al. 1997; Kim et al. 2001; Li and Zhou 2001). Recent findings have shown that activation of alpha2 adrenoreceptors via the release of norepinephrine from pericellular sympathetic fibres modulates the excitability of large rat DRG neurons (Ji et al. 2022). Sympathetic fibres in DRG are not restricted to baskets. Quantitative ultrastructural analysis of postganglionic sympathetic fibres in guinea pig DRG showed that a small proportion of fibres were associated with intraganglionic blood vessels and some with epithelial cells of the capsule (Kummer 1994). Furthermore, unlike sympathetic nerve fibres that release transmitters *en passant* and act on sensory neurons via volume transmission, motoneurons have been shown to directly synapse on sensory DRG neurons. Studies in cat cervical DRG demonstrated that axon collaterals from a small subpopulation of motoneurons synapse on sensory neurons (Kayahara 1986). Sensory neurons and motoneurons projected to the same target tissue, the neck muscles. The exclusively axo-somatic synapses were present predominantly on large neurons and contained round and dense-cored vesicles, neurofilaments, and mitochondria. The synapses were asymmetrical with thicker postsynaptic membranes and presynaptic membranes that were associated with electrodense material (Kayahara 1986; Kayahara et al. 1981).

## Classification of DRG neurons based on their ultrastructure

Sensory neurons of DRG and trigeminal ganglia can be subdivided into different subtypes based on functional, immunohistochemical characteristics or unique mRNA expression patterns (Avraham et al. 2021; Klein et al. 2021; Mapps et al. 2022; North et al. 2019; Ray et al. 2018). The diversity of sensory neurons can be extended into their ultrastructural characteristics.

**Fig. 1:**
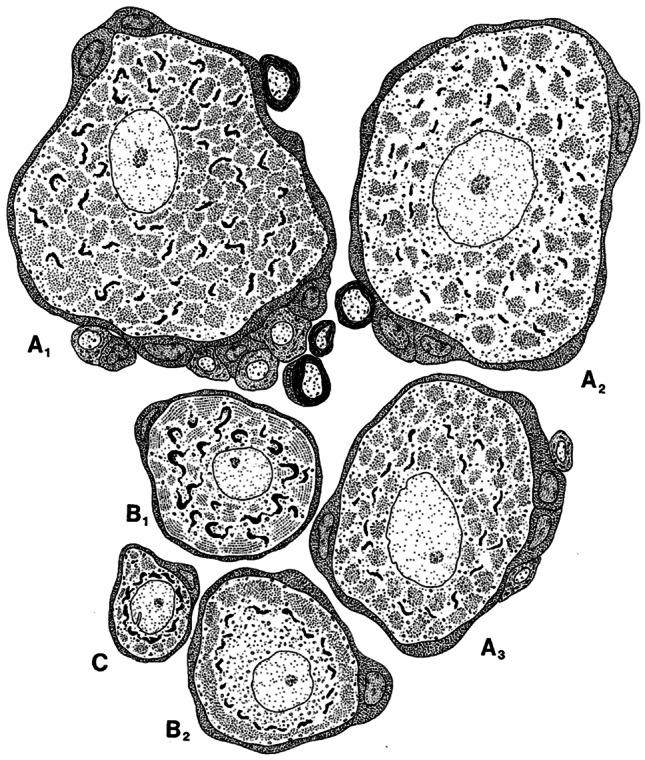
Illustration from Rambourg et al. (1983), outlining the characteristics of six types of dorsal root ganglion neurons (A1–3, B 1 and 2, C) based on ultrastructural features (see Table 1). The structure and localisation of the rough endoplasmic reticulum and Golgi apparatus were mainly used for differentiation between subtypes

**Fig. 2:**
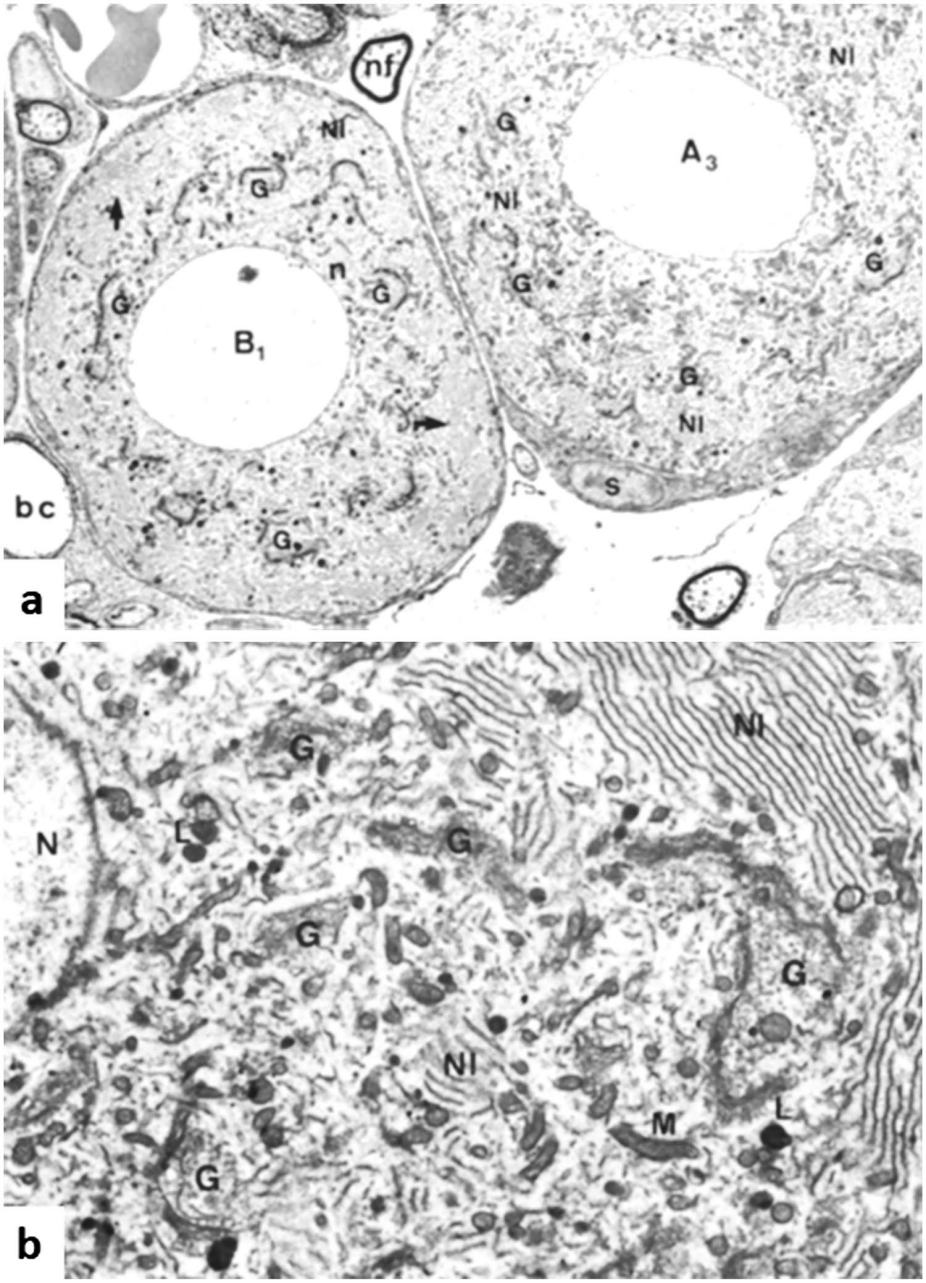
Electron micrographs of osmium-stained thin sections of rat DRG (Rambourg et al. 1983). **a** Two subtypes of DRG neurons, B1 and A3 (see Table 1). The neuronal profile indicated with B1 presents Golgi apparatus (G) that localises as a perinuclear ring with the rER (NI) that is present in outer part of the soma. The soma indicated with A3 shows that the rER and Golgi apparatus are interspersed throughout the profile (nf, nerve fibre; bc, blood capillary; s, satellite cell, magnification × 3000). **b** Cytoplasm of the B1 subtype in higher magnification with parallel arrangement of the ER (NI) whereas the Golgi apparatus (G) and a proportion of mitochondria (M) are found perinuclear. Also present are lysosomes (L) and part of the nucleus (N) with a dark stained nuclear membrane (magnification × 9500)

Early light-microscopic investigations observed that nerve cells in the ganglia did not appear to represent a homogeneous population. Consequently, they were divided into “small dark” and “larger light” neurons (Koneff 1887), which were named type A (light) and type B (dark) cells respectively (Hossack 1954). Based on electron microscopic investigations (Dawson et al. 1955; Hess 1955), Andres confirmed the presence of more than one type of sensory neurons in rat DRGs (Andres 1961a). He further subdivided each light (type A) and dark (type B) cell type into three subtypes, A_1-3_ and B_1-3_, based on morphological and ultrastructural differences, such as size and the structure of the nucleus and cytoplasm, myelination of neurites, and the distribution of the rough ER (Table 1) (Andres 1961a). Subsequent ultrastructural investigations identified a third small cell type, “type-C cells” in rat DRG and expanded the cell subtypes into seven. In those studies, similar to the initial investigations by Andres, differences among cell types A, B, and C were based on variations in the location, amount, and structure of organelles such as the Golgi apparatus and ER, ribosomes, and neurofilaments in the cytoplasm (Table 1) (Duce and Keen 1977). These studies not only confirmed ultrastructurally the presence of different types of sensory neurons, but also their random distribution across the margins of the DRG. Specific types of neurons were not confined to specific regions within a ganglion. In addition, the concept that subtypes of each cell type represented different functional states of a parent cell type was tested by electrically activating DRGs and ultrastructurally investigating the cells. Findings indicated that cell subtypes did not represent different functional states of parent cells (Duce and Keen 1977), which further confirmed the presence of different sensory neuron types. Improvements in fixation techniques led to the re-evaluation of sensory neuron subtypes in DRG based on the location and morphology of cell organelles, as before. According to these findings, Rambourg et al. (Malchiodi et al. 1986; Rambourg et al. 1983) subdivided rat DRG sensory neurons into three categories with a total of six subtypes, A_1–3_, B_1,2_, and C (Figs. 1, 2 and Table 1). This basic subdivision into three categories was later confirmed in laboratory animals and recently in human DRG by methods such as multiple labelling immunohistochemistry and spatial transcriptomics (Cavanaugh et al. 2011; Klein et al. 2021; Ray et al. 2018). Transcriptomics and single-cell sequencing further subgrouped neurons and defined up to 12 different neuron subtypes (Mapps et al. 2022; North et al. 2019; Ray et al. 2018).

Neuron subtypes mentioned above were identified in rat DRG, but ultrastructurally diverse neuron subtypes have been identified in several other species. In mice, seven subtypes of neurons, Aα–γ, Bα–γ, and C, were identified using ultrastructural characteristics in combination with histochemical detection of carbonic anhydrase and acid phosphatase as well as the determination of [3H]L-glutamine uptake and peptide content (Sommer et al. 1985) (Table 1). In the opossum, the traditional two types of neurons, small dark and light were distinguished. In the cytoplasm of the small dark neurons, mitochondria and neurofilaments were dispersed throughout the cell, while the Golgi apparatus and ER were perinuclear and at the periphery of the cell, respectively. Light neurons showed a more homogenous distribution of organelles across the cytoplasm with more prominent bundles of neurofilaments (Soares et al. 2012). In cats, light microscopically DRG cells could not be clearly separated into large light and small dark cells, but ultrastructurally larger somata showed more organised rER compared to the smaller cells (Johnson and Sears 2013). Chicken DRG neurons were classified into large light A-type and smaller dark B-type neurons and further subclassified into four subtypes A_1–2_ and B_1–2_ (Philippe and Droz 1988) (Table 1). Frog neurons were also classified as large light A-type cells and dark B-type cells (Berthold 1966). Dorsal root ganglion cells of Italian wall lizard *Podarcis sicula* were ultrastructurally differentiated into four types of neurons, one small dark B-type cell and three subtypes of larger light A-type cells (Geuna et al. 1998). The data indicate that the ultrastructure of DRG neurons varies within ganglia but also between species. The structural data are supported by recent functional data, highlighting difference in the expression of voltage-gated ion channels between rat and human DRG neurons (North et al. 2019; Zhang et al. 2017).

## Immuno-electronmicroscopical findings on DRG neurons

Immuno-electron microscopy (immuno-EM) has been employed in the classification of sensory neuron subtypes using their neurochemical characteristics. Larger rat type-A neurons showed localisation of anti-alpha2-adrenoreceptor antibodies on intra-cytoplasmatic membranes as well as the plasma membrane suggesting interaction with adrenoreceptor ligands such as noradrenaline (Shinder et al. 1999). Immuno-EM investigation of chick DRGs has revealed the presence of prostaglandin synthase D in chick B _1_ type sensory neurons (Philippe and Droz 1988; Vesin and Droz 1995). The discovery of the gaseous neurotransmitter, nitric oxide (NO), led researchers to identify the cells that produced the gas with the NADPH-diaphorase reaction product as a marker for the identification of cells containing nitric oxide synthases (NOS). The ultrastructure of guinea-pig DRG neurons was examined using immuno-EM to investigate whether NADPH-diaphorase and NOS shared the same location in cells. In this work, the presence of the reaction product in neurons was compared with the presence of NOS protein using immuno-EM. Findings indicated that the NADPH-diaphorase product was associated with membranes of many organelles, while the NOS immunoprecipitate was predominantly present in the cytoplasm, thus not allowing clear-cut conclusions. Furthermore, this study did not investigate the presence of NOS or NADPH-diaphorase activity in A- or B-type cells (Zhou et al. 1998). Subsequently, Henrich et al. demonstrated that both the NADPH-diaphorase reaction product and the endothelial NOS isoform at the cisterns of the smooth endoplasmic reticulum adjacent to mitochondria in all DRG neurons (Henrich et al. 2002). The study also showed the presence of neuronal NOS at juxtamitochondrial junctions as well outside of junctions in neuronal NOS positive neurons. Immuno-EM also revealed the presence of receptors for nerve growth factor not only in the cytosol of DRG neurons but also satellite cells (Pannese and Procacci 2002). A recent investigation of human DRG demonstrated connections containing connexins 37, 43, and 45 between developing human sensory neurons, indicating the presence of gap junctions between neurons (Juric et al. 2020).

## Structure of satellite cells

The dorsal root ganglia contain two main types of glial cells, Schwann cells and satellite cells. Satellite cells are closely associated with the cell bodies of neurons. The name “satellite cells” for cells that envelope sensory neurons was chosen by Cajal in 1909, and this was accepted in publications that described the ultrastructure of satellite cells using electron microscopy in the 1950s–1960s (Anderson and Van Breemen 1958; Cervos-Navarro 1959; Hess 1955; Pannese 1960). During this period, it was unclear whether satellite cells were interconnected, if they completely sheath neuronal somata, or if pores that bypassed the satellite layer allowed direct communication between neuron and interstitial space. Several subsequent investigations have shown that individual DRG neurons of different species were normally completely surrounded by satellite cells (Cervos-Navarro 1959; Pannese 1960; Rigon et al. 2013; Ruiz-Soto et al. 2020). Scanning EM analysis showed that the neuron-satellite complexes in frogs and chickens have diameters of about 25 μm respectively (Matsuda et al. 2005). However, there are species-specific exceptions. In mice, electron microscopy showed that 70% of DRG neurons are surrounded by individual sheaths of satellite glia, whereas in 30% of cases, groups of 2–6 neurons are covered by one satellite glia cell sheath (Blum et al. 2014). This covering of several neurons by one sheath was seen both in situ and in cultured DRG explants (Bunge et al. 1967). In rats and cows, there can be more than one layer of satellite cells surrounding a neuron, and two neurons can share one satellite cell capsule (Andres 1961a; McCracken and Dow 1973).

**Fig. 3:**
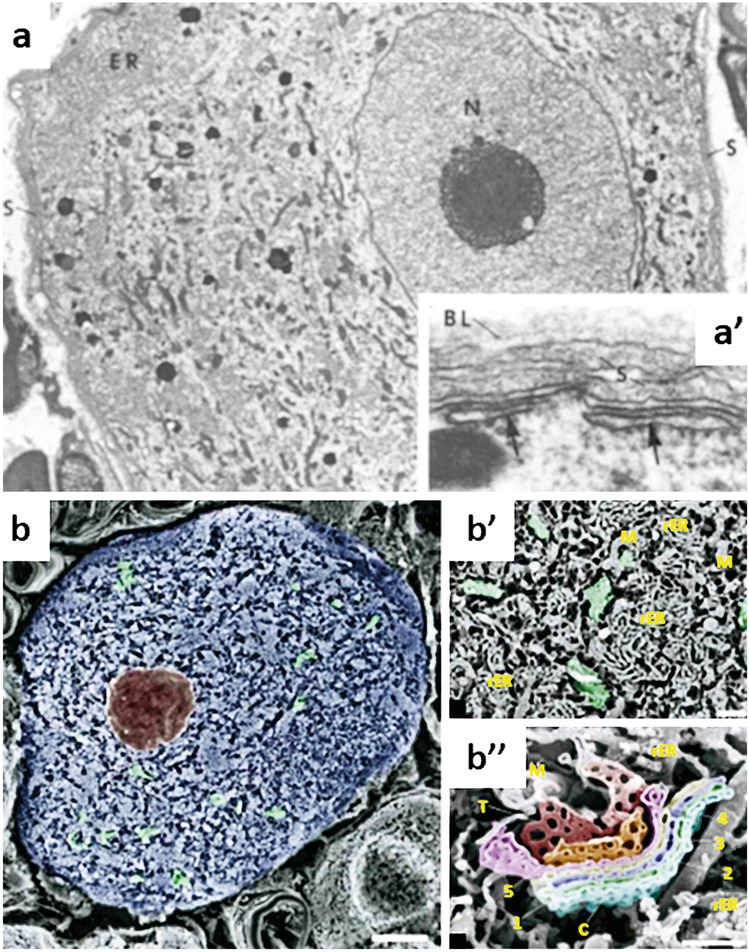
**a** Transmission electron microscopy image of an osmium-stained thin frog DRG section. It shows the neuronal profile of an unclassified DRG neuron. The ER is present in the outer margin of the soma, whereas the Golgi apparatus, lysosomes, and mitochondria seem to be localised around a nucleus (N) with a prominent nucleolus. The satellite cell surrounds the soma (magnification × 6000). Inset **a’** demonstrates the close relationship between cisterns (arrows) which might be part of the rER and the plasma membrane and the neuronal plasma membrane and satellite cells. A basal lamina (BL) covers the satellite cell only at the external side (magnification × 90,000). Images used with permission from Matsumoto and Rosenbluth (1985). **b** Pseudo-coloured scanning electron microscopy image of an osmium-macerated rat DRG (Koga and Ushiki 2006). DRG neuron with Golgi apparatus indicated in green. The Golgi stacks are dispersed throughout the cytoplasm. Bar 6 mm. Inset **b’** indicates the close relationship between Golgi stacks (green) and abundant surrounding rER and mitochondria. Bar 1 mm. Inset **b’’** shows a Golgi stack in high magnification with Cis- (C) and Trans- (T) Golgi which are indicated. The five present cisterns are indicated. Bar 500 nm.

Sometimes the satellite cell layer is very thin (150 nm). The external surface of satellite cells is covered with a 10–20-nm-thick basal lamina that separates satellite cells and cells outside of the neuron-satellite complex (Andres 1961a; Cervos-Navarro 1959) (Fig. 3) that continues alongside Schwann cells (Andres 1961a; van den Bosch de Aguilar and Vanneste 1983). Usually, the basal lamina is separated from the satellite cell by a 20-nm-wide space (Table 2). Connections between satellite cells contain tight junctions (van den Bosch de Aguilar and Vanneste 1983) and gap junctions (Blum et al. 2014), which have been confirmed in developing human DRGs by the presence of connexins 37, 43, and 45 (Juric et al. 2020). There is no basal lamina between a satellite cell and a neuron. Transmission EM studies have shown that the thickness of the plasma membrane of satellite cells was 7–12 nm in rats and frogs and had many folds and borders. In addition, the neuronal cell body is separated from the satellite cells by a 10–12 nm wide intercellular space (Andres 1961a; Rigon et al. 2013).

The processes of satellite cells overlap and interdigitate with neurons. The zone between satellite cells and neurons is characterized by interdigitating membrane extensions from both cells, although the neuronal surface seems to be more ruffled compared to the adjacent satellite cell plasma membrane (Bunge et al. 1967). However, there are no gap junctions between a satellite cell and a neuron (Blum et al. 2014). Interestingly, in dogs, interdigitations between satellite cells and neurons have been shown to be connected by desmosomes (Tongtako et al. 2017). The interdigitations might present areas of interaction between satellite cells and neurons which is supported by immune-EM and the demonstration of the enzyme phosphatidylinositol 4-phosphate 5-kinase (PIP5K) in opposing membranes of satellite cells and neurons, which indicates presence of areas enriched in channels and other membrane proteins (Chomphoo et al. 2021).

In addition to interdigitations, there seem to be also gaps in the layer as early described by Cervos-Navarro and Andres (Andres 1961a; Blum et al. 2014; Cervos-Navarro 1959; Li et al. 2011; McCracken and Dow 1973). Rarely, small areas of a neuron not covered from satellite cells and in direct contact with the basal lamina were described (Cervos-Navarro 1959).

## Organelles in satellite cells

### Nucleus/nucleolus

Satellite cells in DRG of mice have round-ovoid nuclei with a diameter of 4–7 μm, a double layer nuclear membrane, and a homogenous nucleoplasm with a peripheral enrichment of heterochromatin (Table 2) (Ruiz-Soto et al. 2020). The chromatin of the satellite cell nucleus appears to be denser compared to neuronal chromatin. Nucleoli are present (Bunge et al. 1967).

### Endoplasmic reticulum

The appearance and amount of the ER seems to be variable. Some authors have described the presence of “well-oriented” cisternae, whereas others identified wider areas in satellite cells that contained ER but did not observe the layered appearance of ER usually observed in nerve cells. Furthermore, it possessed fewer ribosomes (Bunge et al. 1967; Cervos-Navarro 1959; Pannese 1960).

### Golgi complexes, multivesicular bodies, and lysosomes

Satellite cells contain an abundance of organelles, including Golgi complexes, multivesicular bodies, lysosomes, coated vesicles, and other structures such as centrioles and filaments (Bunge et al. 1967; McCracken and Dow 1973; Ruiz-Soto et al. 2020). Vesicles appear to be larger and wider in vitro compared to in situ measurements, but similar to the in vivo situation, vesicles have been described as abundant at the satellite cell-neuron interface (Bunge et al. 1967). They usually have a diameter of 35–70 nm (Andres 1961a; Pannese 1960).

### Mitochondria

Satellite cells commonly possess small mitochondria that lack well-defined cristae. However, the type of mitochondria in satellite cells associated with certain types of neurons may vary. This does not indicate that the neuron types could be identified based on the ultrastructural appearance of the mitochondria in satellite cells (Andres 1961a).

### Microtubules and neurofilaments

Bovine satellite cells only infrequently showed microtubules and microfilaments (McCracken and Dow 1973). Interestingly, the inner layer of satellite cells adjacent to the neuronal surface possesses high amounts of filamentous actin, F-actin, which extends into satellite cell processes. Those projections were invaginated into neuronal folds (Li et al. 2011). The outer layer of satellite cells is bordered by a basal lamina and connective tissues (Blum et al. 2014).

### Lipofuscin

Lipofuscin appears to be not present in satellite cells. Ultrastructural changes that are related to aging, such as lipofuscin granule accumulations in neuronal somata, have been reported in rats as early as 6 months, but have not been observed in satellite cells until 24 months of age (van den Bosch de Aguilar and Vanneste 1983).

## Structure and organelles of Schwann cells

Both Schwann cells and satellite cells originate from the neural crest. But in contrast to satellite cells, Schwann cells surround the sensory neurons processes within the DRG. The number of unmyelinated neurons ensheathed by an individual non-myelinating Schwann cell varies across the DRG with a higher number of axons associated with one Schwann cell located in the distal part of a DRG (Harty and Monk 2017; Murinson and Griffin 2004). In contrast, in areas with a higher density of neuronal profiles, these Remak bundles contain lower numbers of processes. The cytoplasm of Schwann cells between axons is very thin with a thickness of about 50 nm; thus, there is not much space between adjacent axons (Murinson and Griffin 2004). Although Schwann cells surround neuronal processes, the cells are never in contact with the body of the neuronal cell. Organelles such as mitochondria and the ER have structures similar to that of satellite cells. Nuclei of Schwann cells have irregular shapes with homogenous nucleoplasm and a small dense nucleolus (Cervos-Navarro 1959). Satellite cells and Schwann cells are found in close proximity in the initial segment of the axon (Nascimento et al. 2018; Zenker and Hogl 1976).

## A new type of glia in DRG

A new type of glia has been identified in rat DRG, and the ultrastructure and array tomography of the glia have been described using transmission electron microscopy (TEM) and SEM respectively (Koike et al. 2019). These glial cells exhibit immunoreactivity to some markers of satellite glia, but unlike other satellite cells, they were immunoreactive for the p75 neurotrophin receptor (p75^NTR^) (Obata et al. 2006). This type of p75^NTR^ positive glia is located adjacent to myelinating Schwann cells, at the point when the cover of the neuronal process transitions from satellite glia to Schwann cells (Koike et al. 2019). A basal lamina surrounds the p75^NTR^-positive glia, which discriminates the cell from mesenchymal cells lacking basal laminas. The nucleus shows large areas of heterochromatin. The cytoplasm is darker, similar to other glial cells of DRGs and contains the usual organelles such as mitochondria and ER (Koike et al. 2019).

## Structure of connective tissue cells in DRG

In addition to neurons, satellite cells and Schwann cells, DRGs consist of a variety of other cell types such as macrophages, fibroblasts, and pericytes (Avraham et al. 2022; Mapps et al. 2022). Ultrastructural data are absent for almost all of the cell types which consequently associate with a lack of understanding of their integration and of the type of contacts between the diverse cell types.

### Macrophages

Relatively high numbers of macrophages are present in normal DRG. In rats, 4000 macrophages have been estimated to reside in a DRG, many adjacent to satellite cells (Lu and Richardson 1993). Recent investigations using single-cell and spatial transcriptomics of different cell types in DRG including macrophages have revealed the presence different subgroups of macrophages in DRG (Avraham et al. 2021; Mapps et al. 2022). Macrophages in DRG are one of the key cells that can modulate pain signalling (Raoof et al. 2021; Ton et al. 2013), but information on DRG macrophage ultrastructure are lacking (Yu et al. 2020). A rare image of the ultrastructure of macrophages in rat DRG has been shown in relation to the uptake of the tracer horseradish peroxidase (HRP). The substance was immediately removed from extravascular spaces in DRG upon extravasation from intraganglionic blood vessels. Unfortunately, no further analysis of macrophage ultrastructure was included in the report (Jacobs et al. 1976).

### Mast cells

Early studies on bovine DRGs identified mast cells, whereas neither macrophages nor leucocytes could be observed (McCracken and Dow 1973). In the opossum DRG, cells with mast cell morphological characteristics were named but not shown or further described (Soares et al. 2012). However, contrary to these findings, recent transcriptomic profiling studies of cell types in human, rat, and mouse DRG did not describe cells with the characteristics of mast cells (Avraham et al. 2022; Mapps et al. 2022).

### Pericytes

Pericytes showed ultrastructural similarities to endothelial cells with lower numbers of vesicles. A basal lamina separated endothelial cells and pericytes (Andres 1961a; McCracken and Dow 1973). The pericytes contained pinocytotic vesicles on their interstitial side (McCracken and Dow 1973).

### Interstitial cells/endoneural cells

Interstitial DRG cells (in early studies named fibrocytary endoneural cells (Andres 1961a)) have been described next to satellite cells in early publications (Cervos-Navarro 1959). Interstitial cell numbers are low compared to satellite and Schwann cells (Andres 1961a; Cervos-Navarro 1959). These cells do not have contact with nerve cells or their processes. Their nuclei are irregular in shape and have homogeneous fine granular nucleoplasms. The rough ER is well developed and dispersed throughout the cell. Polyribosomes are less frequent compared to neurons, and the plasma membrane is irregular but lacks folds. Long processes of interstitial cells are loosely connected to Schwann cells. No basal lamina separates interstitial cells from the interstitial space (Andres 1961a; Cervos-Navarro 1959). The absence of the basal lamina is also a characteristic feature of interstitial fibroblasts (McCracken and Dow 1973) which suggests interstitial cells are likely to be fibroblasts which have been identified in transcriptomic studies and might be involved in the infiltration of immune cells into DRG in response to nerve damage (Avraham et al. 2022; Mapps et al. 2022; Singhmar et al. 2020).

## Structure of blood vessels and capsule of DRG

### Blood vessels

**Fig. 4:**
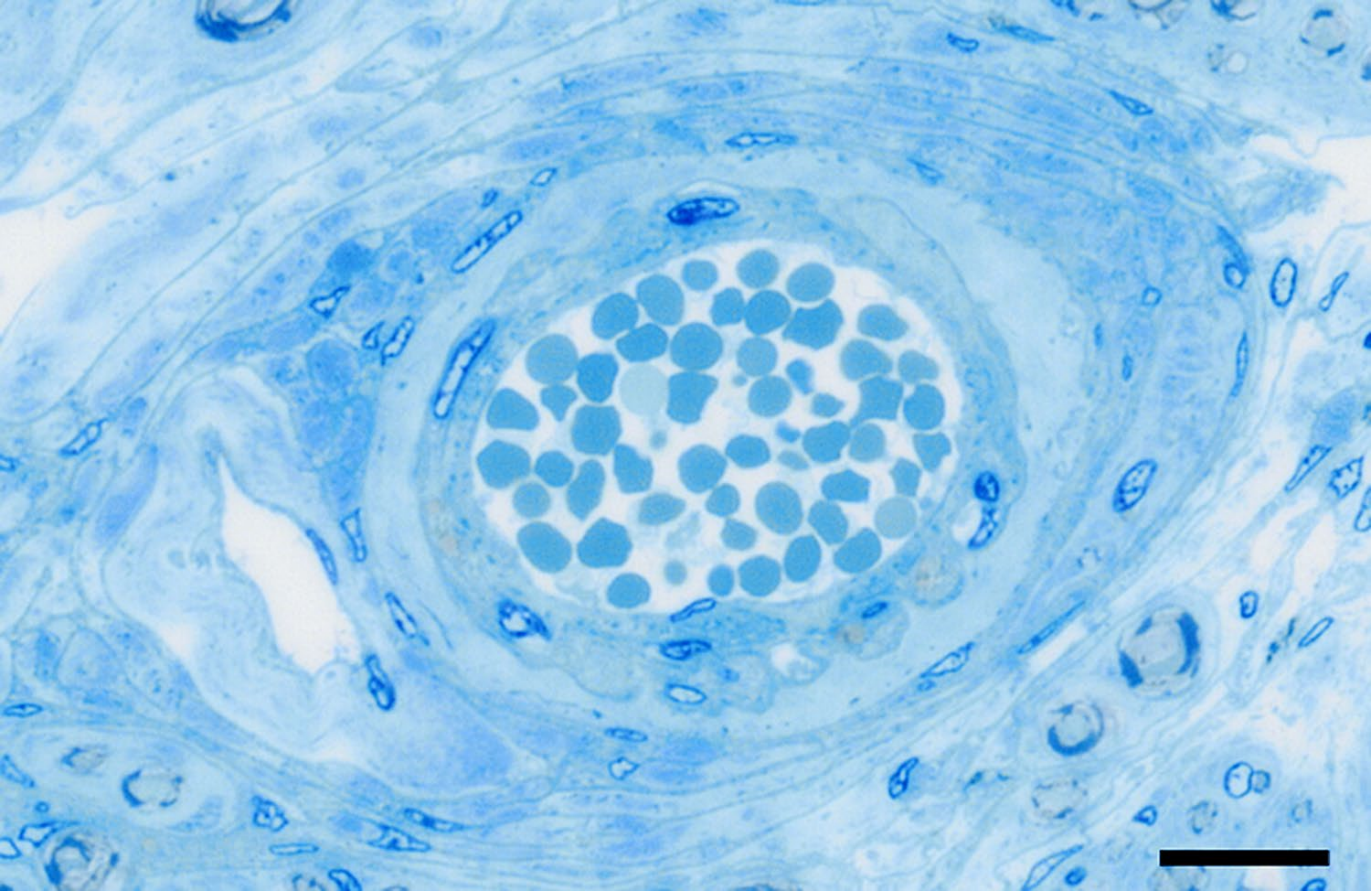
Semithin section of a human DRG. The largest blood vessels in human DRG are small arterioles and venules as shown in the semithin section. Bar 20 mm

The recent COVID-19 pandemic is a multisystem disease partially due to vascular endothelium injury, and the persistence of symptoms in long COVID patients includes chronic pain and sensory abnormalities that are likely to propel new investigative paths to therapeutic opportunities targeting the DRG vasculature. Sensory neurons have long processes and need substantial energy to maintain bidirectional transport of cytoplasmic and membranous contents. This high energy demand requires an adequate blood supply; thus, DRGs are highly vascularised. The three-dimensional arrangement of the vasculature in rat DRG was visualised using corrosion cast technique in combination with SEM (Kobayashi et al. 2010). The largest intraganglionic blood vessels in human DRG are arterioles and small veins (unpublished observation, Fig. 4). Arterioles penetrate the ganglionic capsule and transform into a subcapsular capillary network that surrounds the neurons. Those arterioles showed constricted areas, suggesting the presence of vascular sphincters. The capillaries were 3–5 μm in diameter and became venules with increasing size to presumably become sinusoidal veins (Kobayashi et al. 2010).

Dorsal root ganglia are situated outside of the blood–brain barrier. Capillaries in DRG allow free perfusion of DRGs with blood for efficient exchange of nutrients, gases, and metabolites (Jacobs et al. 1976). The endothelial cell layer has been shown to possess fenestrations with and without a visible diaphragm (Jacobs et al. 1976). This contrasts early investigations, where the capillary endothelium was described as containing no “pores” (Andres 1961a). However, analysis of the distribution of a tracer (HRP) at the ultrastructural level in rats showed capillary fenestrations and fast leakage of tracer into extravascular spaces, before being taken up by macrophages (Jacobs et al. 1976). The capillary network has been shown to surround neuron-satellite complexes. The processes of capillary endothelial cells overlapped and were connected via junctional complexes. The cells were in contact with a 50-nm-thick basal lamina (Andres 1961a).

### Capsule and endoneurium

Dorsal root ganglia are located at the intersection between the central nervous system (CNS) and peripheral nervous system (PNS). Therefore, cell types and structures that differ between the CNS and the PNS, such as the meninges, must change along DRG. Consequently, early studies focused on investigating the relationship between the meninges and the capsule of DRG (Brierley 1950). Brierley summarized early experiments that started in the 1870s (Quincke 1872, Key and Retzius 1875), and based on experiments in rabbits, concluded that the dura and arachnoid layers converge with the capsule of ganglia and create a cul-de-sac at the narrowing of the proximal DRG (Brierley 1950). The subdural space then continues along the ganglion accompanied by the transition of the dural layers of the CNS into the epi- and perineural structures associated with DRGs (McCabe and Low 1969). The capsule of the rat, bovine, and opossum DRGs has been described in early electron-microscopical investigations as consisting of small, flattened cells and layers of collagen fibres (Beams et al. 1952; McCracken and Dow 1973). These cells are covered from derivates of the dura mater or, as described in the epineurium, continue as dura mater (McCabe and Low 1969). The fibrous capsule contains a space covered by “endothelial” cells (Andres 1961a). The perineurium follows as layers of perineural epithelial cells that have elongated nuclei and a rough ER that is less developed compared to fibroblasts (McCracken and Dow 1973). Glycogen granules and pinocytic vesicles are frequently present (McCracken and Dow 1973). It is separated from the collagen- and fibroblast-rich epineurium and endoneurium by basal laminas (McCabe and Low 1969; McCracken and Dow 1973). The layers of flattened epithelial cells and spaces filled with connective tissue surround the subarachnoid space (McCabe and Low 1969; Soares et al. 2012).

The endoneurium contains collagen fibril aggregations that extend from the fibrous capsule. Fibrocyte-like cells are present, but low in numbers compared to satellite cells (Andres 1961a; Cervos-Navarro 1959). They possess a rough ER that contains pores (Andres 1961a). Interestingly, fibrocyte-like cells are not separated from other endoneural cells by a basal lamina. The endoneural cells contain lipid droplets and large cytosomes, which are lysosome-related organelles.

## Summary

The identity and relevance of the dorsal root ganglia have evolved from being seen as rudimentary structures housing cell bodies of sensory neurons to highly organised signal relay and modulation complexes that are essential for the transmission of sensory information related to our internal and external environment. The unique pseudounipolar structure of each DRG neuron has one axon that bifurcates into two separate branches resulting in a proximal process and a distal process which can be some of the longest cell extensions in the body. Another unique property of DRGs is that they lie outside the blood–brain barrier but inside the vertebral column covered in part by meninges (Haberberger et al. 2019). The DRG has significant clinical relevance in nerve injury, inflammation, and chronic pain development. Our understanding of the complexity of the cellular environment within the DRG has expanded over the last five decades, and the DRG is increasingly investigated as a key component in the development of chronic pain conditions and a potential target for new therapies. Therefore, comprehensive knowledge about the ultrastructure of the DRG and its components at the cellular level is essential to gain a better understanding of structure–function relationships. This knowledge will be vital for many of these new emerging therapies to reach their projected potential. The ultrastructural data generated to date is largely focused on investigating sensory neurons situated within the DRG. More recent studies have pivoted towards understanding functionally important interactions involving the non-neuronal cells within DRG that possess the capacity to modulate DRG neuron function. The described heterogeneity in ultrastructure between sensory DRG neurons (Andres 1961a; Duce and Keen 1977; Matsuda and Uehara 1981; Rambourg et al. 1983) has recently be confirmed by using gene-deficient mouse models and RNA sequencing (North et al. 2019; Wangzhou et al. 2020). Nevertheless, currently available evidence exposes the existence of a major deficiency between the understanding of the pathophysiology of illnesses such as chronic pain gained by animal research and existing human research data. This is clearly highlighted by the low animal-to-human clinical translation rates and has been reflected to date in the disappointing inadequacy of pharmacological treatment development for most chronic pain conditions, while direct targeting of DRG has recently been demonstrated to overcome some of the limitations of systemic approaches. Neurostimulation of the DRG to relieve chronic neuropathic pain is an example of beneficial effects of current progressive research (Deer et al. 2017). Understanding of similarities and differences between animal and human DRGs at an ultrastructural level is imperative in the translation of animal research for direct therapeutic approaches involving the human DRG. Therefore, it is essential to focus not only on DRG neurons, but also on other cells such as macrophages and satellite cells, that appear to play important roles in the modulation of pain perceptions. What impact may this have on DRG therapies targeting pain? Ultrastructural investigations in this area should focus on integrating structural investigations with functional relationships of macrophages with satellite cells and the presence and cell–cell interactions of other immune cells such as T and B immune cells. In resolving some of the issues with therapeutic targeting of DRG, it is also important to gain a better understanding of the blood supply of DRG and associated structures. Dorsal root ganglia are highly vascularised structures; however, the knowledge about the DRG blood supply and migration and presence of immune-competent cells as well as the presence of lymphatic vessels within ganglia has been the subject of very few investigations or has not been investigated at all.

A deeper understanding of the ultrastructure of the DRG should provide the critical information necessary to address important clinical challenges related to the function of the DRG. Virus infections such as varicella zoster virus and SARS-cov-2 all impact DRG functions, and understanding the mechanisms of pathogenesis electron microscopy and related techniques (e.g. immuno-EM, histochemistry) are vital. These investigations may, in turn, provide valuable insights into potential new therapies. Gene therapy uses viral vectors to gain access to target cells (Wagner et al. 2021). It is crucial to understand cells, their connections, and their interactions with a virus at the ultrastructural level. Furthermore, intercellular communication via exosomes between macrophages and DRG neurons has also been shown to regulate pain signalling (Simeoli et al. 2017). Exosome therapy is being developed as a highly targeted, flexible treatment for conditions involving the DRG, including osteoarthritis and chronic pain (Miao et al. 2021; Shiue et al. 2019). In addition, insights into cell–cell interactions may reveal DRG cell type–specific targets to alleviate chronic pain resulting from pathological conditions. Therefore, in-depth knowledge of the ultrastructure of DRG, particularly in humans, will help to “see through” the framework of cell-to-cell functional relationships in these principal structures of the PNS.These in turn may help to identify one of the crucial keys that could unlock the underlying mechanisms that regulate the primary stages of sensory signalling in both healthy and diseased states.
